# Entrepreneurial Profiles at the University: A Competence Approach

**DOI:** 10.3389/fpsyg.2020.612796

**Published:** 2020-12-18

**Authors:** Sofía Louise Martínez-Martínez, Rafael Ventura

**Affiliations:** Faculty of Economics and Business, University of Malaga, Málaga, Spain

**Keywords:** entrepreneurship, Entrepreneurial University, entrepreneurial competences, competence profiles, grit, social capital

## Abstract

The Entrepreneurial University plays a central role in entrepreneurial ecosystems and actively influences the development of entrepreneurial human capital, which is a critical asset for many economies. There is thus a requirement for the identification and strengthening of entrepreneurial competences, but no previous studies have included any analysis of these competences in the university context using an approach based on profiles. The present study fills this gap by investigating the existence of different entrepreneurial profiles among students, based on their competences. It also defines key competences that are critical for differentiating between these profiles and improving entrepreneurial competence levels more generally. To meet these objectives, a field research campaign was developed. Data on 1104 students from various degrees and faculties were collected and analyzed using a quantitative methodological approach. The results reveal the existence of four entrepreneurial competence profiles, namely *low profile*, *top profile*, *social profile*, and *grit profile*. Among as many as 12 possible entrepreneurial competences, the most prominent can explain to a large extent the entrepreneurial profiles of students; these are networking and professional social skills, community engagement, perseverance of effort, and consistency of interest. The results provide evidence of the importance of social capital and *grit*. In addition to their contribution to the theory in this area and the development of the Entrepreneurial University paradigm, the results are also useful for the design of training strategies aimed at strengthening the levels of competence of students, thereby providing universities with tools to foster the creation of entrepreneurial human capital.

## Introduction

Business and entrepreneurship ecosystems, defined by the collaborative creation of new value and a critical participation in entrepreneurial initiatives, have increasingly been noted to be of service in integrating approaches used to achieve disruptive innovation and improve performance. In this sense, universities can be considered as ecosystems, and they too require a disruptive innovative perspective in order to face the challenges placed on them by society. In this context, the concept of the “Entrepreneurial University” has emerged, referring to a university’s evolution toward an ecosystem that combines teaching, research, and knowledge transfer to favor the development of entrepreneurial initiatives with social and economic value (Gibb and Hannon, 2006; Guerrero et al., 2014; Ventura and Quero, 2017). The Entrepreneurial University involves the implementation of radical innovation to change the traditional conception of the institution (Ventura et al., 2019). Audretsch (2009) highlights the importance of knowledge-based entrepreneurship, stating that “entrepreneurial activity is the missing link between investments in new knowledge and economic growth” (p.27).

For a university to be truly entrepreneurial, the promotion of entrepreneurship must be carried out from a systemic point of view, with a clear orientation toward innovation and the dissemination of an entrepreneurial culture (Kirby, 2002). Isenberg (2011) points to the importance of policy strategies regarding the setup of the ecosystem, and places special emphasis on the value of the inherent human capital. Specifically, human capital with the capacity to be entrepreneurial has a key and determining role regarding the outcomes of a disruptive innovation system. Training in entrepreneurial activity, especially at higher levels throughout the university system, is thus considered an essential ingredient when increasing the entrepreneurial capital resource of an economy. The entrepreneurial university must therefore place special emphasis on fomenting entrepreneurial human capital, by developing competences that enable the setting up of new projects (Audretsch, 2014).

According to Chiru et al. (2012), the term “competence” refers to a combination of knowledge, tools, values, and attitudes that allow effective and efficient personal or professional performance. Based on this concept, entrepreneurial competences are those that enable the creation and discovery of opportunities in the environment and their use in a company’s establishment and successful management (Hunjet et al., 2015). Numerous investigations have been carried out to define the term and establish different categorizations of competence. However, entrepreneurial competence has received scant attention regarding the university environment, and no previous work is known to have addressed the entrepreneurial competence profile of university students. In order to fill this perceived gap in our understanding, we conducted empirical research based on the following research questions:

Q1: Is the university population heterogeneous in terms of entrepreneurial competences, and is it possible to identify different entrepreneurial profiles among university students based on their competences?

Q2: Are there key competences that are critical to differentiating between competence profiles and improving the competence levels of students?

To answer these questions, we present the results of a bibliographic review of previous research on the definition and nature of the competences needed for entrepreneurial activity. The study adopts the classification proposed by Morris et al. (2013), who identify 13 competences and developed a questionnaire geared to their measurement. Based on 1104 student responses, we use a quantitative methodology to analyze the entrepreneurial competences and the profiles that permit differentiation between groups of students by referring to the most developed skills or core competences. The measurement of competence allows us to reach a conclusion on the entrepreneurial competences of the university population, which provides new knowledge to improve teaching and learning. This will lead to the better acquisition and development of entrepreneurial competences in the university ecosystem, which will in turn foster entrepreneurial human capital, thus enhancing disruptive innovation and the Entrepreneurial University.

The structure of the remainder of this article is as follows. First, we stress the importance of Entrepreneurial Universities, as well as their potential to generate well qualified human capital through the development of entrepreneurial competences. Then, we discuss the definition of competences, presenting different views and categorizations. We present the empirical methods and results of our work, and finally we discuss the theoretical contributions together with the educational and entrepreneurial implications.

## Theoretical Framework

### The Entrepreneurial University as a Provider of Entrepreneurial Human Capital

Universities can play a crucial role in the outcomes of innovation ecosystems, given the importance of knowledge-based entrepreneurship as a catalyst for economic development and job creation (Audretsch, 2009). In this respect, universities provide a liaison between industry and government, laying the foundations for the proliferation of relationships based on innovation (Etzkowitz and Leydesdorff, 2000). The relevance of the university is based mainly on its potential to generate new knowledge, as well as providing entrepreneurial and well-qualified human capital (Zahra and Wright, 2011; Castellacci and Natera, 2013; Guerrero et al., 2016). As Audretsch (2014) indicates, universities must condition the supply of an economy’s entrepreneurial capital, directly affecting business creation and entrepreneurial dynamics. In this context, the paradigm “Entrepreneurial University” acquires special relevance, and constitutes the theoretical framework of this study. This conception of university, first introduced by Etzkowitz (1983), focuses on the influence of the university on the environment and on the related interactions involved in encouraging progress and development. Specifically, in the knowledge society universities have a challenging role in becoming organizations that are more socially and economically relevant (Nelles and Vorley, 2011).

When the concept was first posited, it referred mainly to universities with a clear focus on innovation, entrepreneurial culture, and a proactive tendency to facilitate knowledge transfer to society through the creation of businesses (Clark, 1998; Kirby, 2002). Knowledge transfer is thus seen as a “third leg” of income generation, distinct from teaching and research. The level of implementation of this third leg conditions the contribution of universities to socio-economic development. Entrepreneurial Universities promote the commercialization of the research results they generate (Jacob et al., 2003; Williams, 2003), seeking new sources of funding in order to encourage investment in university entrepreneurship (Yokohama, 2006). Moreover, the Entrepreneurial University is also characterized by the design of new spaces and services that facilitate the creation of companies based on technology and knowledge (Chrisman et al., 1995; Etzkowitz, 2003).

That said, the purpose of the Entrepreneurial University has evolved over time to transcend this third-leg, knowledge-transfer mission, both by developing entrepreneurial activity and by fostering the entrepreneurial behavior of the institution as a whole. In this respect, the Entrepreneurial University adds to the entrepreneurial culture in the management of the institution, involving all agents in the creation of an entrepreneurial ecosystem, one that is interconnected with its environment and where new relationships are generated between university community agents and between the institution and companies (Friedman and Silberman, 2003; Etzkowtiz, 2004; Rizzo, 2015). Through its mediating role, entrepreneurial universities catalyze creativity and knowledge and favor exchange of information between the actors in the ecosystem (Mele and Russo-Spena, 2015). In the words of Sam and Van der Sijde (2014) “an Entrepreneurial University actively identifies and exploits opportunities to improve itself (with regard to education and research) and its surroundings (knowledge transfer) and is capable of managing (governing) the mutual dependency and impact of the three university tasks” (p. 902). The Entrepreneurial University implies a constant interchange between the educational institution and the rest of society, involving and engaging different actors.

In words of Fantauzzi et al. (2019), there are three key aspects of the university paradigm: strategic and operational decision-making to create connections with the environment, connections with the agents of the environment, (e.g., with other institutions or companies), and the entrepreneurial attitudes and actions of those who make up the university (teachers, researchers, and students). This last aspect is closely linked with the development of the entrepreneurial competences of these actors. Following a bibliometric review, Skute (2019) highlights the existence of four different approaches in the study of the Entrepreneurial University, namely partner complementary, ecosystem, interaction channel, and academic entrepreneurship. The present research is framed within this last theoretical approach, which focuses on the characteristics of academic entrepreneurs and their engagement in business creation (D’Este et al., 2012). It highlights the relevance of entrepreneurial competences, experiences, perceived norms, and intentions to undertake entrepreneurial initiatives, as well as the mechanisms that promote this entrepreneurial human capital.

The fostering of entrepreneurial human capital in the university, through the generation, attraction, and retention of entrepreneurs, is one of the main objectives of the Entrepreneurial University (Bramwell and Wolfe, 2008). The education and development of entrepreneurial students encompasses both tangible and intangible aspects, such as the acceptance and image of the entrepreneur in society, the existence of sufficient economic resources to meet the financial needs of the initiatives, and above all, a strong training in entrepreneurship (Ventura and Quero, 2017). Human Capital is defined by Becker (1993) to be a set of competences, knowledge, abilities, and skills acquired through education and training, such that the design of a high-quality entrepreneurial education based on the development of competences is key to achieving this primary objective of the Entrepreneurial University. In this respect, the identification and definition of the entrepreneurial competences of the students are crucial for generating an increasingly entrepreneurial form of human capital. The following section is a theoretical review of the concept and existing classifications, which frames the study of competence as developed here.

### Entrepreneurial Competences

In recent decades, the development of competence has been studied extensively in numerous disciplines, including psychology (Sternberg and Kolligian, 1990), education (Burke, 1989), human resources (Burgoyne, 1993), and business organization (Boyatzis, 1982). Competences are complementary and independent aspects of these subjects and can be used in different fields (Rey, 1996). The diversity of disciplines that address the study of competence and the plurality of contexts in which they are applied makes the definition of the term particularly complex.

Several terms are used in the scientific literature to refer to the concept of competence: “skills,” “expertise,” “acumen,” and “competency” (Mitchelmore and Rowley, 2010; Arafeh, 2016). These terms make reference to abilities, capabilities, capacities, qualifications, and other related attributes (Baartman et al., 2007). Such terminological diversity makes international consensus on the subject difficult, in both academic and applied fields, hindering the development of common knowledge and expressions that could lead to a connection between the research initiatives and their practical applications (Mitchelmore and Rowley, 2010). Jubb and Robotham (1997) state that it remains a challenge to develop a widely accepted definition of competences to foster common ground between researchers and trainers. Likewise, Boon and Van der Klink (2003) hold that competence remains a “fuzzy concept.” Although several decades have passed since its first conceptualization, there is still a great terminological diversity in this area of knowledge. Even so, the existence of shared characteristics in the different conceptual approaches is evident.

The most common factors referred to as “competence” are personal ability, knowledge, and having the tools necessary to achieve personal or professional goals. The European Parliament and Council (2006) explains competence as a combination of skills, knowledge and attitudes. Chiru et al. (2012, p. 4011) define it as the proven ability to “select, combine and use the appropriate knowledge, skills and other acquisitions (values and attitudes) in order to successfully solve a particular category of work or learning situations and for professional or personal development in terms of effectiveness and efficiency.” The definition of Morris et al. (2013, p.353) follows the same logic, indicating that a competence “refers to the knowledge, skills, attitudes, values, and behaviors that people need to successfully perform a particular activity or task.” According to Wynne and Stringer (1997), competences are what people need to develop to achieve the outputs required for their job, referring to what they know, do, and think. Along the same lines, Mertens (1996) relates competence to an individual’s capacity to achieve a particular goal in a given context.

It is also important to highlight that the term “competence” influences both the personal and the professional sphere. In this sense, the European Qualification Framework has established that competence is a “proven ability to use knowledge, skills and personal, social and/or methodological abilities in work or study situations and in professional and personal development” (European Parliament and Council, 2008, p. 4). In this sense, numerous efforts have been made by various institutions and in academia to define models of competence that help to explain professional behaviors, performances, and outcomes (Schippmann et al., 2000; Kurz and Bartram, 2002; Sanchez and Levine, 2009). An example of theoretical and practical development in this area is the metamodel created by Bellini et al. (2019) on the objectives proposed by the European Qualification Framework (European Commission, 2005). However, such models do not focus on the competences needed for self-employment and entrepreneurship, but rather on competences for employment and professional success within a company from the perspective of human resources. Therefore, in order to contribute to the paradigm of the Entrepreneurial University, a complementary approach is considered necessary, to focus specifically on the entrepreneurial competences.

Competences, especially those that foster entrepreneurial capacities, are crucial for the development of entrepreneurial human capital. Cubico et al. (2010) indicate that there are certain personal qualities that distinguish between non-entrepreneurs and entrepreneurs, and condition the business success of the latter. The competences are not only understood as key to the professional development of individuals, but also to their personal growth. In this sense, numerous studies explain entrepreneurial competences as transversal aspects that influence various spheres of life and foster active participation in society (Bacigalupo et al., 2016). In 2006, the published “Recommendation on key competences for lifelong learning” highlighted that the “sense of initiative and entrepreneurship,” understood as the capacity to turn ideas into action, is a key competence for all citizens (European Parliament and Council, 2006). Therefore, based on the importance of entrepreneurial education for the progress of society, the European Commission developed an “Entrepreneurship Competence Framework or EntreComp” to promote a common understanding of the entrepreneurial competences.

Entrepreneurial competences also have strong implications for business creation and activities related to the entrepreneurial process. The development of an entrepreneurial project is strongly influenced by the levels of competence and profiles of those who participate in it. The self-awareness of these levels is also relevant, because this facilitates communication and increases the professional autonomy of the entrepreneurs (Bellini et al., 2019). The personal characteristics of entrepreneurs, and their knowledge, skills, and experiences are key strategic resources for organizations and have a positive impact on business success (Lewis and Churchill, 1983; McClelland, 1987; Barney, 1991; Kiggundu, 2002; Onstenk, 2003). It is therefore important to extend both the study of entrepreneurial competences and the analysis of entrepreneurial competence profiles in order to understand the degree to which entrepreneurial competences are the result of individual or contextual factors (Gümüsay and Bohné, 2018), and to detect the key competences needed to develop entrepreneurial human capital. The aim, in other words, is to identify critical primary competences that stand out for their relevance or for their need for reinforcement. These should be at the core of the design and implementation of training programs, given their importance for the success of such programs (Burke, 1989; Voorhees, 2001; Onstenk, 2003).

Al-Mamun et al. (2016) define entrepreneurial competences as the skills needed to use resources to improve the performance of a micro-company. For Boyatzis (1982), competence is a person’s capacity to meet the job demands of a certain business environment to reach desired results. Mitchelmore and Rowley (2010) refer to the set of competences that operationalize a venture in a company, both technical and non-technical (Huck and McEwen, 1991). Similarly, Hunjet et al. (2015) define entrepreneurial competence as:

A combination of knowledge, skills, attitudes and capabilities to create and discover opportunities in the environment, to introduce changes, and to direct one’s behavior toward successful creation and management of an organization, whose purpose it is to take advantage of these opportunities and to deal with a high level of uncertainty and complexity in a challenging environment (p. 623).

From the various definitions of entrepreneurial competences reviewed here, it is possible to identify certain common characteristics. In this sense, the entrepreneurial competences are considered to be individual capacities, in terms of a set of knowledge, expertise, skills, tools, attitudes, and values oriented to reach professional development and to achieve entrepreneurial goals. They are also treated as important aspects in the successful performance of entrepreneurial activities in terms of effectiveness and efficiency, meeting the entrepreneurial demands of society. Based on these shared characteristics, the present study considers entrepreneurial competences as knowledge, experiences, skills, and attitudes, which enable and favor the success of entrepreneurial activities.

The structuring of entrepreneurial competences into coherent groups “has proven to be challenging, due to the interconnected and multifaceted character of entrepreneurship as a competence” (Komarkova et al., 2015, p. 71). Nevertheless, many attempts have been made by public institutions and in academia to determine some classifications of entrepreneurial competences. EntreComp, developed by the European Commission (Bacigalupo et al., 2016), builds a competence model in which 3 areas and 15 specific interrelated and interconnected entrepreneurial competences are identified. In particular, “‘Ideas and opportunities,’ ‘Resources,’ and “Into Action” are the 3 areas of the conceptual model and they have been labeled to stress entrepreneurship competence as the ability to transform ideas and opportunities into action by mobilizing resources” (p.10). This European benchmark is used to distinguish the following entrepreneurial competences: spotting opportunities, creativity, vision, valuing ideas, ethical and sustainable thinking, self-awareness and self-efficacy, motivation and perseverance, mobilizing resources, financial and economic literacy, mobilizing others, taking the initiative, planning and management, coping with uncertainty, ambiguity and risk, working with others, learning through experience.

In the same vein, several authors have offered classifications that serve to identify the competences related to entrepreneurial activity. Table 1 presents different categorizations of competence according to the author concerned, together with the number of competences and their characteristics. It can be seen that while the number of identified competences varies, there are similarities in their characteristics.

**TABLE 1:** Classifications of entrepreneurial competences according to different authors.

Authors	N°	Entrepreneurial competences
Hayton and Kelley, 2006	4	Innovation, intermediation, defense, sponsorship
Chandler and Jansen, 1992	2	Ability to recognize and seize opportunities, willingness and capacity for intense effort.
Di Zhang and Bruning, 2011	5	Market orientation, entrepreneurial orientation, need for achievement, internal locus of control, need for cognition
Abdullah et al., 2009	8	Progress, achievement orientation, commitment, decision-making capacity, risk management, tenacity, networking, optimism
Man et al., 2002; Kaur and Bains, 2013	6	Opportunity competence, relationship competence, conceptual competence, organizing competence, strategic competence, commitment competence
Onstenk, 2003	3	Ability to recognize and analyze market opportunities, ability to communicate and detect attitudes, to persuade and discuss with stakeholders, capacity for networking and learning effectively from business interactions.
Wu, 2009	23	Analytical thinking, business acumen, customer orientation, commitment to learning, communication, conceptual thinking, order and quality, developing others, empathy, expertise, flexibility, influence, information seeking, initiative, innovation, organizational awareness, personal motivation, relationship building, results orientation, self-confidence, self-control, team leadership, verbal and written communication.
Morris et al., 2013	13	Opportunity recognition, opportunity assessment, risk management, conveying a complete vision/vision of the future, tenacity/perseverance, creative problem solving/creativity, resource leveraging, guerrilla skills value creation. New products, services and models, ability to maintain focus and adapt, resilience, self-efficacy, networking and social skills

## Materials and Methods

In order to achieve the stated objectives and answer the research questions raised, a quantitative methodology is developed to analyze the entrepreneurial competences of a population of university students. We then outline the data collection and methodology used for the analysis.

### Data Collection

The classification of competence used here as the key reference is that developed and validated by Morris et al. (2013). These authors identified 13 entrepreneurial competences using a two-sample, three-round Delphi approach method, through which industry experts, consisting of entrepreneurs and entrepreneurship training professionals, worked together to compile a list of entrepreneurial skills. Instrumental reliability was corroborated using pre-/post-testing. The effectiveness of this methodology for reaching consensus has been demonstrated when panels of experts are used (Chan et al., 2001). The instrument used to measure the 13 competences is a questionnaire of 111 items on a five-point Likert scale. Table A1 (additional material) shows the classification of entrepreneurial competences and the student assessment questionnaire used, as developed by Morris et al. (2013).

The sample was composed of 1104 students from 52 Bachelor’s and Master’s degrees in 16 different faculties of the University of Malaga, Spain; 36.1% (*n* = 399) of the sample were male and 63.9% were female (*n* = 705). The questionnaire was completed online between October 2019 and April 2020 within the framework of the student’s registration on the university employment platform Talentank. There is some diversity in the sample regarding the origins of the qualifications and the number of academic years completed. According to Liñán and Chen (2009), studies based on a population of university students offer the advantages of homogeneity and similarity in terms of age and qualifications.

### Analyses

Using Stata version 14.0, a twofold multivariate approach was chosen to determine the existence of entrepreneurial profiles among the university students in the sample. First, we employed an exploratory factor analysis (EFA) with a rotation procedure to identify the underlying dimensions of the entrepreneurial competences of the population (Bachhaus et al., 2011) and to reduce the number of variables. After testing different methods of EFA and rotation procedures with similar results, the principal factors were selected with an orthogonal varimax rotation. The number of retained factors with this method is consistent with previous literature on the subject using the same measurement instrument (Morris et al., 2013) and the different factors are clearly defined through the item scores. Following the recommendations of Hair et al. (1998), we included only factor loadings greater than 0.3, and the variables clearly loaded in the different factors. In the present study, the sample size is greater than 1000, a condition considered excellent by MacCallum et al. (1999). The Kaiser–Meyer–Olkin (KMO) was used to validate the adequacy of the sample for factorial analysis, and having obtained the factors, we measured internal consistency using Cronbach’s alpha.

Second, we performed a cluster analysis. After testing various methods of hierarchical and non-hierarchical clustering with similar results, we determined that the clearest grouping was provided by Ward’s Hierarchical agglomerative method with a squared Euclidean measure of distance. We used the generated factors as variables, to divide the sample into homogenous groups, and to determine the entrepreneurial competence profiles of the university students and the possible differences between them. Two stopping rules, recommended for hierarchical clustering, were applied to determine the optimal number of groups, namely the Caliński and Harabasz (1974) and the Duda et al. (2001). For the former, larger values of pseudo-F indicate more of a distinction between clusters, while in the latter, larger values of Je(2)/Je(1) and small pseudo-T-squared values are more convenient for the definition of the appropriate number of clusters.

We verified the normal distribution of the variables using different graphical methods according to sample size (Histogram, Stem and Leaf diagram, and Kernel Density test), and analyzed the differences between groups. First, an ANOVA test was carried out for each competence considering the cluster variable as grouping variable. Subsequently, an ANOVA test was applied for pairs of clusters, to facilitate the interpretation of the competence profiles.

## Results

Having detected and eliminated outliers, the database contained 1081 cases. The results of the correlation analysis and EFA demonstrated the need to eliminate some items that did not fit well within the scales, due to insufficient correlation with the other items of the matrix (i.e., <30) (Pett et al., 2003). Use of the factor analysis technique to identify latent factors was validated. The sample was considered adequate with a KMO of 0.9330 for all variables and a significance of 0.000 from Bartlett’s test of Sphericity.

The common factor model of EFA allowed the initial number of 111 variables to be reduced, leaving a total of 12 factors. These were retained according to the information provided by the Scree test (Catell, 1966) and the Kaiser criteria, and based on the eigenvalues or amount of variance of the items accounted for by a factor (Norris and Lecavalier, 2010). All the extracted factors had eigenvalues > 1 (Table 2), and these 12 factors explained 97.74% of the total variance. After ensuring that the extracted factors were not correlated, we applied an orthogonal rotation (varimax) to simplify the configuration of the factors and enhance their interpretability (Browne, 2001). The rotated factor loadings are shown in Table A2 (additional material). Having shown that the internal consistency of the factors was high with a Cronbach’s alpha of greater than 0.7 in most cases, the competences could be interpreted according to the different factor loadings.

**TABLE 2:** Retained factors of EFA, method: principal factors, rotation: orthogonal varimax (n° of factors: 12; eigenvalues > 1; explained variance: 97.74%).

Factor	Variance	Difference	Proportion	Cumulative
Factor 1	5.64783	0.72006	0.1514	0.1514
Factor 2	4.92777	0.92413	0.1321	0.2836
Factor 3	4.00364	0.49332	0.1074	0.3909
Factor 4	3.51032	0.04418	0.0941	0.4851
Factor 5	3.46614	0.36234	0.0929	0.5780
Factor 6	3.10380	0.92300	0.0832	0.6612
Factor 7	2.18081	0.04436	0.0585	0.7197
Factor 8	2.13644	0.12455	0.0573	0.7770
Factor 9	2.01189	0.09125	0.0539	0.8309
Factor 10	1.92064	0.05536	0.0515	0.8824
Factor 11	1.86528	0.18694	0.0500	0.9324
Factor 12	1.67834		0.0450	0.9774

*Prob > chi2 = 0.0000.*

The 12 factors correspond to competences included in the classification of Morris et al. (2013). Some of these competences are identified by specific factors, while others are now subdivided into more than one factor. This is the case for value creation with new products, services, and business models, and tenacity/perseverance. The first of these is divided into value creation through observation/experimentation and value creation through questioning, while the second is split into perseverance of effort and consistency of interest. Table 3 shows the identification of each factor according to items with higher loadings, its Cronbach’s Alpha, mean, and standard deviation. The entrepreneurial competences of the university population are then described through the interpretation of the items loaded in each factor.

**TABLE 3:** Entrepreneurial competences and descriptive characteristics.

Factor	Ítems	Reliability (Cronbach’s Alpha)	Mean	Std. Dev.
Factor 1: Networking and professional social skills	p13a, p13b, p13c, p13d, p13e, p13f, p13g, p13h, p13i, p13j, p13k, p13l	0.8841	−1.50e-09	0.9280179
Factor 2: Creativity	p6a, p6b, p6c, p6d, p6e, p6f, p8a	0.8726	−4.23e-10	0.8975807
Factor 3: Value creation through observation and experimentation	p9g, p9h, p9i, p9j, p9k, p9l, p9m, p9n	0.8607	4.33e-10	0.8948328
Factor 4: Value creation through questioning	p9a, p9b, p9c, p9d, p9e, p9f, p8b	0.8378	3.14e-10	0.9129371
Factor 5: Risk management and environmental control	p3a, p3b, p3c, p3d, p12b, p12d	0.5379	1.49e-10	0.9160554
Factor 6: Opportunity assessment	p2a, p2b, p2c, p2d, p2e, p4a, p4b	0.8210	4.92e-10	0.8850492
Factor 7: Bootstrapping and resource management	p7i, p7j, p7k, p7l, p7m, p7n	0.7196	8.64e-11	0.8496865
Factor 8: Perseverance of effort	p5f, p5g, p5h, p5i, p5j	0.7479	−2.17e-10	0.8500921
Factor 9: Opportunity recognition	p1b, p1c, p1d, p1e	0.6403	4.87e-10	0.8426086
Factor 10: Consistency of interest	p5a, p5b, p5c	0.7596	−6.91e-10	0.8547087
Factor 11: Community engagement	p13m, 13n, 13o, p13p	0.7382	−2.10e-10	0.8655921
Factor 12: Resilience	p10e, p10f, p11a, p11b, p11c	0.7430	−7.58e-10	0.8221428

(1) Networking and professional social skills. Competence that enables the establishment, development and maintenance of relationships with others to obtain work and career advantage (Forret and Dougherty, 2001). Networking is related to career outcomes such as income and promotion (Burt, 1992). The social capital created through the networking competence provides valuable information, resources, and opportunities. Individuals can use their networks to achieve entrepreneurial goals and advantages in terms of business competitiveness (García and Valencia, 2009).

(2) Creativity. Ability to create novel, original, unexpected, and useful outcomes through the relationship between previously unrelated objects (Sternberg, 1999; Lee et al., 2004). Creative thinking is an important element in problem-solving and decision-making, and fosters entrepreneurial intention (Hamidi et al., 2008). Thus, creative individuals are more likely to start and engage in entrepreneurial projects (Ward, 2004).

(3) Value creation through observation and experimentation. Ability to develop new products, services, and/or business models by observation and experimentation. Both observation and experimentation are considered crucial to the development of innovation and entrepreneurial initiatives (Mulder et al., 2007). The behavioral approach to entrepreneurship highlights the importance of what the entrepreneur does (Gartner, 1989). In this sense, an entrepreneurially oriented individual searches for information through non-verbal scanning and seeks experiences that enable innovation and the identification of new opportunities (Kaish and Gilad, 1991; Dyer et al., 2008).

(4) Value creation through questioning. Capability of developing new products, services, and/or business models by questioning the *status quo* and people’s fundamental assumptions (Morris et al., 2013). In this sense, the information obtained serves to facilitate and improve the decision-making process. It is related to the concept of entrepreneurial curiosity, which is known to be a motivational system oriented toward investigation, i.e., an interest in novelty and a tendency to search for answers to learn tasks related to entrepreneurship (Jeraj, 2014). This curiosity is positively related to entrepreneurial value creation, fostering the generation of business ideas (Peljko et al., 2016). Cubico et al. (2010) relate curiosity to innovation, which in turn is defined by them as an entrepreneurial aptitude.

(5) Risk management and environmental control. Ability to handle uncertainty and reduce hazards and the potential impact of the risk if it occurs. It also involves the ability to control and shape the environment (Morris et al., 2013). This locus of control is related to the conception of self-efficacy and is fundamental to the development of entrepreneurial intentions (Krueger et al., 2000). Risk and environmental handling are considered crucial to the decision-making process of an entrepreneur, who must deal with unexpected situations and conflicting information (Zimmerer and Scarborough, 2002).

(6) Opportunity assessment. Ability to analyze the extent to which a recognized opportunity is viable and can provide competitive advantage. This is an evaluation of the content structure of opportunities in order to determine their attractiveness and decide whether they represent a business opportunity with a potential profit (Tang et al., 2012; Morris et al., 2013). It is thus related to opportunity recognition (see below). It is an important entrepreneurial ability that affects the decision-making processes used by entrepreneurs. Effective evaluation of the circumstances involved may result in improvement of the entrepreneurial initiative through the integration of both intangible (e.g., new knowledge or processes) and tangible (e.g., new products) resources (Haynie et al., 2009).

(7) Bootstrapping and resource management. Ability to access to resources and extract value from them. This also includes the ability to recombine and seek new ways of obtaining resources when these are limited (Politis et al., 2011). In this regard, the importance of the bootstrapping concept is that it refers to the development of methods to ensure the use of the resource at low or no cost. In this way, the resources need not necessarily be owned (Winborg, 2009). A resource is a tangible or intangible asset that is available and can be used for entrepreneurial purposes (Davidsson, 2005), therefore the acquisition and use of these resources determines the success of entrepreneurial initiatives (Politis et al., 2011).

(8) Perseverance of effort. Ability to persevere and sustain efforts to achieve intended objectives even when hardships or setbacks occur (Salisu et al., 2020). Along with consistency of interest (see below) it represents one of the two dimensions of grit, a psychological concept that is positively correlated with success and the achievement of long-term objectives (Duckworth et al., 2007). It is linked to the competence of perseverance described by Morris et al. (2013), and positively connected to entrepreneurial sucess. It is positively related to entrepreneurial success in that a persevering attitude is required to face the difficulties and obstacles related to the creation and development of ventures (Mooradian et al., 2016; Salisu et al., 2020).

(9) Opportunity recognition. Competence that encompasses both the recognition of links between trends, changes, and events that appear to be unconnected, and the pattern recognition behind these connections (Baron, 2006). It is based on the willingness to access information and requires a state of alertness, i.e., an ability to identify opportunities overlooked by others (Kirzner, 1979). Opportunity recognition is the catalyst of entrepreneurial activity (Dyer et al., 2008). According to Shane and Venkataraman (2000), there is no entrepreneurship without opportunity.

(10) Consistency of interest. Ability to stay focused and passionate over a long period of time by performing a particular task without changing interest or goals (Salisu et al., 2020). Along with perseverance of effort, this is the other of the two dimensions of grit (Duckworth et al., 2007; Arco-Tirado et al., 2018), and it is again related to the perseverance described by Morris et al. (2013). It is an important competence for entrepreneurship in that the creation of venture implies complex, multiple, and competing objectives whose scope requires a maintained focus over long periods of time.

(11) Community engagement. Social interaction that fosters participation in communities through the establishment, development, and maintenance of social relationships with different groups (Morris et al., 2013). Engagement in different communities provides a great diversity of social capital, which is considered positive for entrepreneurial initiatives. Thus, the existence of formal and informal networks within the social structure can enhance entrepreneurial activities and reduce their cost (Portela and Neira, 2002). Belonging to different communities provides a greater access to information and enhances the recognition of opportunities (Granovetter, 1995). Entrepreneurs therefore usually have a higher diversity of contacts in their networks than non-entrepreneurs (Renzulli et al., 2000).

(12) Resilience. Ability to adapt to environmental changes in situations of threat, adversity, tragedy, trauma, or stress whilst maintaining a positive mindset (Salisu et al., 2020). It is related to the ability to transform a situation of adversity into an enjoyable challenge (Greitens, 2015), and is an important competence both for the entrepreneurial initiative and for the sustainability of ventures over time, determining entrepreneurial success (Fisher et al., 2016). Individuals who run businesses need a resilient attitude to overcome numerous setbacks, e.g., financial shortfalls, which can occur especially in times of crisis (Pal et al., 2014).

**FIGURE 1:**
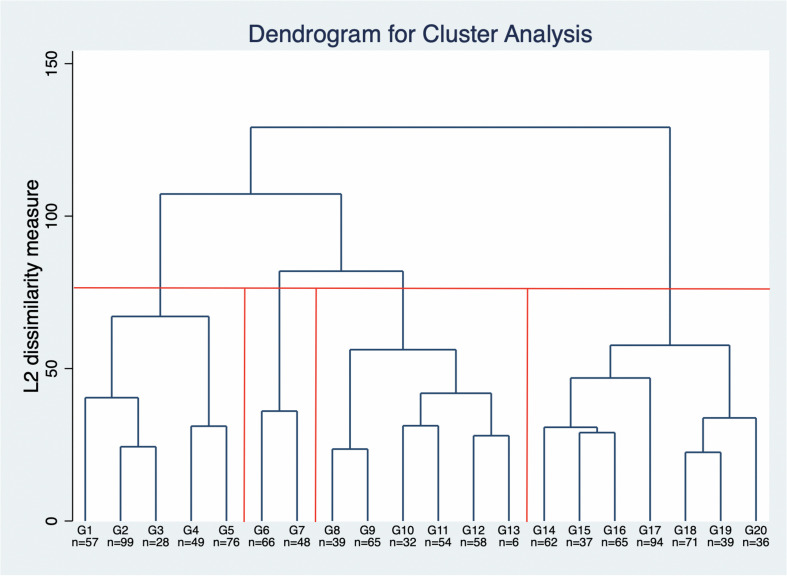
Truncated cluster-dendrogram of Ward’s Hierarchical agglomerative method. The red line shows the four-cluster-solution representing the different competence profiles of university students.

The hierarchical cluster analysis developed to identify homogenous groups in the sample, considering the 12 identified competences as variables, shows evidence of different competence profiles. Both Caliński-Harabas and Duda-Hart’s Je(2)/Je(1) stopping rules point to an optimal solution of 4 clusters. The truncated dendrogram (Figure 1) shows a visual interpretation of the grouping.

Table 4 shows the number of cases and the mean of the 12 entrepreneurial competences by cluster.

**TABLE 4:** Number of cases and mean of the entrepreneurial competences by cluster (total *n* = 1081).

	Cluster 1	Cluster 2	Cluster 3	Cluster 4
*n*	309	114	254	404
%	28,6%	10,5%	23,5%	37,4%
1. Networking and professional social skills	−0.322	0.458	0.517	−0.208
2. Creativity	−0.088	0.242	0.250	−0.158
3. Value creation through observation and experimentation	−0.149	0.345	−0.063	0.056
4. Value creation through questioning	−0.163	−0.005	0.133	0.042
5. Risk management and environmental control	−0.831	0.253	−0.220	0.703
6. Opportunity assessment	0.057	0.434	−0.074	−0.119
7. Bootstraping and resource management	−0.350	0.299	0.042	0.157
8. Perseverance of effort	−0.200	0.268	−0.138	0.164
9. Opportunity recognition	−0.006	0.153	0.261	−0.202
10. Consistency of interest	0.199	0.736	−0.670	0.061
11. Community engagement	−0.190	1.099	0.220	−0.304
12. Resilience	0.195	0.269	−0.164	−0.121

**FIGURE 2:**
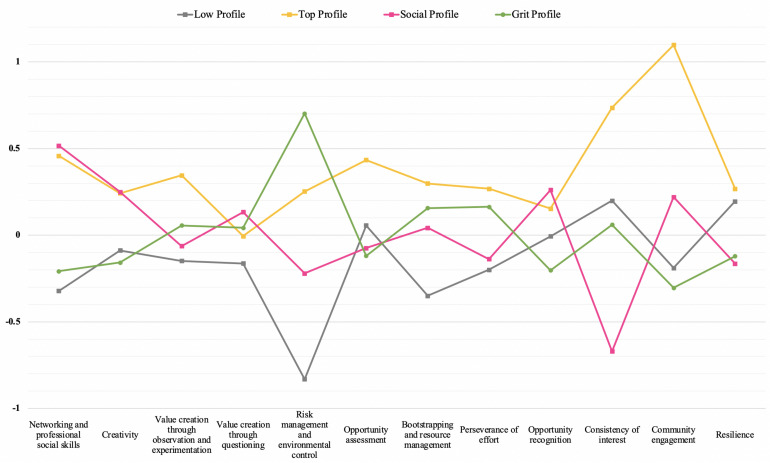
Different entrepreneurial profiles found for university students. The *x*-axis shows the 12 entrepreneurial competences used to determine the profiles. The *y*-axis shows the means of standardized scores (+1 denotes one standard deviation better than the average sample score).

The information provided by the statistical tests together with the average values for each competence by cluster, as expressed graphically in Figure 2, allows the interpretation of the competence profiles and yields the following results.

Profile 1: Below-average scores for most competences. Low levels of development of key competences for entrepreneurial practice, in particular poor networking and professional social skills, bootstrapping and resource management, or risk management and environmental control. The best scores for this profile are found in the competences of resilience and consistency of interest. Opportunity assessment is also somewhat more developed than in two of the other three profiles. Nevertheless, low levels for the remainder of the entrepreneurial competences indicate that although people in this profile may recognize opportunities and may have the ability to work on their goals over a long period of time, with a positive mindset that facilitates adaptation to environmental changes, they nevertheless lack the necessary tools to launch an entrepreneurial project and develop it successfully. Thus, given the low scores in both absolute and relative terms, this profile can be referred to as the *low entrepreneurial profile*.

Profile 2: This profile is characterized by high scores in all twelve entrepreneurial competences, which are all more pronounced than they are in the other profiles. The students in this second group have highly developed social skills in both professional and community settings. In this sense, their engagement in community projects is considerable. They know how to evaluate opportunities and generate value from them, more through observation and experimentation than by asking questions. They are also those best able to manage resources when these are limited. Aside from their outstanding social skills, the competences linked with psychological attributes are also highly developed. They are resilient, persevering, and above all consistent in their interest, being able to maintain focus on long-term objectives and develop entrepreneurial projects in a sustainable way. This cluster is therefore called the *top entrepreneurial profile*.

Profile 3: Members of this group stand out in certain entrepreneurial competences, with above-average scores. However, this profile is also characterized by deficiencies in other ways. Students with this profile present a high level of social skills, as for Profile 2. However, this group is characterized by the tendency to develop them in a professional context, rather than in community settings. They also have high competence scores in opportunity recognition, creativity, and in creating value by asking questions, as opposed to the previous profile, which is characterized by higher levels of observation and experimentation. By contrast, they have lower levels of competence in areas linked to management. In this sense, students in this profile are less successful in developing competences of experimentation, opportunity assessment, resource management, or risk management and environmental control. The greatest competence deficiency of students in this group is found in their low levels of perseverance, consistency of interest, and resilience. These last two competences are linked more closely to psychological factors, and here they present levels below the other three groups. This profile is nevertheless potentially entrepreneurial in that it relates to the gathering of relevant competences to start and develop an entrepreneurial project, for example opportunity detection, creativity, networking and communication. Due to the outstanding professional social skills of this group this profile can be defined as the *social entrepreneurial profile*.

Profile 4: This profile also brings together important entrepreneurial competences, yet it shows deficiencies in others. Students belonging to this group show below-average networking and professional social skills. These scores are more than half a point below those of profiles 2 and 3. Opportunity recognition, opportunity assessment, and creativity are also underdeveloped competences in this group, with averages below those in the other profiles. Even so, these students stand out for above-average levels of competence in other areas such as value creation, bootstrapping, resource and risk management, and control of the environment. They are persevering people with consistency in their interests, in which they score better than those in profile 3. These results show complementarity with profile 3, in that neither group has high levels in all the entrepreneurial competences, but the areas of greatest deficiency in one are the most developed in the other, and vice versa. Thus, while profile 3 stands out in terms of social skills, detection of opportunities and creativity, profile 4 is characterized by highly developed managemental skills and *grit*-related competences. For this reason, the fourth profile can be termed the *grit entrepreneurial profile*.

Several statistical tests were carried out to validate these results and determine the existence of significant differences between profiles. First, the graphical tests of normality show the normal distribution of the twelve competences, which allows the use of ANOVA tests. The results show evidence of differences in all competences (*p* < 0.005), which corroborates the identification of 4 distinct profiles. The ANOVA tests for pairs of profiles show significant differences between profiles (Table 5). The *low entrepreneurial profile* contains differences with the other profiles in most of the competences. The differences between the other groups lie in the competences used for their definitions. In this sense, the *social entrepreneurial profile* is similar to the *top profile* in networking and professional social skills, creativity, value creation through questioning, and opportunity recognition. In the same way, the *grit entrepreneurial profile* presents similarities with the most developed profile in perseverance of effort. The results shown by the ANOVA tests reinforce the complementarity of the *social* and *grit* profiles. This pair of profiles present the biggest differences in their critical competences. In this sense, the competences related to social capital (community engagement and networking/professional social skills) and to the *grit* construct (perseverance of effort and consistency of interest) present the highest statistical differences (significance level: 0.000), demonstrating the importance of these concepts in the definitions of entrepreneurial competence profiles.

**TABLE 5:** Significant differences of competence by pair of profiles (low, top, social, and grit).

		*Top*	*Social*	*Grit*
1. Networking and professional social skills	*Low*	0.0000***	0.0000***	0.1005
	*Top*		0.4765	0.0000***
	*Social*			0.0000***

2. Creativity	*Low*	0.0008***	0.0000***	0.3198
	*Top*		0.9293	0.0001***
	*Social*			0.0000***

3. Value creation through observation and experimentation	*Low*	0.0000***	0.2438	0.0052**
	*Top*		0.0000***	0.0027**
	*Social*			0.0769*

4. Value creation through questioning	*Low*	0.1140	0.0000***	0.0049**
	*Top*		0.1220	0.6524
	*Social*			0.2083

5. Risk management and environmental control	*Low*	0.0000***	0.0000***	0.0000***
	*Top*		0.0000***	0.0000***
	*Social*			0.0000***

6. Opportunity assessment	*Low*	0.0000***	0.0727	0.0082**
	*Top*		0.0000***	0.0000***
	*Social*			0.5294

7. Bootstrapping and resource management	*Low*	0.0000***	0.0000***	0.0000***
	*Top*		0.0034**	0.0844
	*Social*			0.0651

8. Perseverance of effort	*Low*	0.0000***	0.3950	0.0000***
	*Top*		0.0000***	0.2222
	*Social*			0.0000***

9. Opportunity recognition	*Low*	0.0606	0.0002***	0.0014**
	*Top*		0.2590	0.0000***
	*Social*			0.0000***

10. Consistency of interest	*Low*	0.0000***	0.0000***	0.0152*
	*Top*		0.0000***	0.0000***
	*Social*			0.0000***

11. Community engagement	*Low*	0.0000***	0.0000***	0.0200*
	*Top*		0.0000***	0.0000***
	*Social*			0.0000***

12. Resilience	*Low*	0.3688	0.0000***	0.0000***
	*Top*		0.0000***	0.0000***
	*Social*			0.5245

***p < 0.001, **p < 0.01, *p < 0.05.

## Discussion

University students are a heterogeneous population in terms of entrepreneurial competences, therefore they present different levels of development in the 12 identified competences. Furthermore, four entrepreneurial competence profiles are identified, which leads to an answer in the affirmative to Q1 (Is the university population heterogeneous in terms of entrepreneurial competences and is it possible to identify different entrepreneurial profiles among university students based on their competences?) Heterogeneity is a positive feature of the population, since the diversity of competences enriches entrepreneurial activity and leads to improved performance. In other words, to meet the great variety of challenges and unforeseen events of the entrepreneurial process, multidisciplinary entrepreneurial teams with different competences are recommended (Weisz et al., 2010).

Considering that only one of the profiles presents uniformly low levels of entrepreneurial competences, it can be said that almost three quarters of the population has entrepreneurial potential. Thus, 71.4% of the students have a *top*, a *social* or a *grit entrepreneurial profile*, implying that they have entrepreneurial competences with which they could start and/or develop a business, although in some cases it would be necessary to strengthen certain competences. The *top entrepreneurial profile*, characterized by high levels in all the competences, comprises 10.5% of the population. These students have exceptional resources for entrepreneurship and stand out for their social skills and their *grit* development, a feature related to psychological capital (Contreras and Juárez, 2013). These competences are also the most differentiating elements in the other two potential entrepreneurial profiles: the *social* and the *grit profile*, each named on the basis of these competences. Even if all 12 competences are important for the design and practice of entrepreneurial activities, we identify four competences that are critical in understanding the diversity of profiles and their complementary nature, namely networking and professional social skills, community engagement, perseverance of effort, and consistency of interest. We can therefore answer in the affirmative for Q2 (Are there key competences that are critical to differentiating between competence profiles and improving the competence levels of students?). With reference to the definitions given in the theoretical review, which indicate that the concept of competence consists of both innate and acquired characteristics, it can be seen how the highlighted competences are related more to levels of personal ability than to developed or acquired knowledge or tools (Morris et al., 2013). As confirmed by Chiru et al. (2012), they are connected to values and attitudes, and depend on personal traits.

The first two competences mentioned above stand out and are key to the definition of the *social profile*. Thus, students belonging to this group have higher levels of social capital, a concept that has been related broadly to entrepreneurial activity and success. Social capital is understood to be a determining factor of economic growth. “The existence of formal and informal networks within the social structure can enhance many activities and make them less costly, which implies having a capacity for better development” (Portela and Neira, 2002, p.31). Social capital is determined by the relationships and resources that emerge in a network, which actors can access by being immersed in it (Nahapiet and Ghoshal, 1998). The network of contacts is one of the most valuable and determining tools for entrepreneurial activities, and becomes especially relevant in the early stages of a project (Van de Ven et al., 1984). The strategic relationships established through networking encourage the creation and sharing of knowledge, and also promote the development of other skills. It can thus be explained why profiles characterized by developed social skills and community engagement are also accompanied by high levels in other competences. In the case of the two entrepreneurial competence profiles with higher social skills (the *top profile* and the *social profile*), both creativity and opportunity recognition are prominent. This could be a result of the information flows of the social networks, given that these represent the ways in which opportunities are recognized (Vohora et al., 2004). In the same way, the exchange of information leads to learning, through which creativity and innovation are enhanced, competitiveness is improved, and consequently entrepreneurship is promoted.

The other competences identified as critical to the development of entrepreneurial human capital form the *grit* construct and characterize the so-called *grit profile*. These competences are considered to be predictors of entrepreneurial behavior (Arco-Tirado et al., 2019), which explains the importance of this group of students. *Grit* is defined as the maintenance of effort and interest to achieve challenges and long-term objectives (Duckworth et al., 2007), and has a positive relationship with innovation and performance in entrepreneurial environments (Mooradian et al., 2016). People with a greater consistency of effort and greater perseverance of interest are thus more likely to opt for entrepreneurship as a career choice (Wolfe and Patel, 2016), and to reach higher levels of entrepreneurial performance, exhibiting a greater commitment to work (Eskreis-Winkler et al., 2014). These characteristics also enhance an individual’s knowledge and growth (Dweck, 2010).

*Grit* is the fuel for entrepreneurship and self-employment (Arco-Tirado et al., 2019), and its two dimensions must therefore be enhanced throughout higher education. Specifically, in relation to young adults, Wolfe and Patel (2016) identify a positive effect of *grit* in the fostering of self-employment, since the qualities of passion and perseverance contribute to counteract limitations associated with age, such as the difficulty of accessing human, social, and financial resources. This is the case for students with a *grit profile*, who show strong perseverance but low levels of social capital in their competences. The fostering of perseverance and consistency of interest over time also has a positive effect on self-efficacy. Development of *grit* qualities boosts higher levels of self-confidence (Verheul et al., 2012), which in turn increase the ability to manage adverse situations, and to improve the consequent self-perceived ability to succeed. In this way, the development of *grit* can help to increase the acquisition of other competences. It might be useful to take this into account when designing entrepreneurial training programs, especially those intended for students belonging to the low entrepreneurial profile, who need encouragement to develop all their entrepreneurial competences.

This study contributes to both theory and practice. It fulfills a perceived gap in the research on entrepreneurial competences among students, providing a classification of entrepreneurial profiles and highlighting the competences that are key to the differentiation between these profiles, and to improve the levels of entrepreneurial competence of students. From an applied point of view, the results are relevant for university education and knowledge transfer. The ability to differentiate between competence profiles is helpful for the design and development of training programs that foster the acquisition of entrepreneurial competence effectively. In this respect, the identification of competence profiles is a key element in facilitating the achievement of educational and entrepreneurial goals (Alda-Varas et al., 2012). The enhancement of entrepreneurial competences is recommended through education in entrepreneurship, especially at university level (Dickson et al., 2008). The present study also makes a positive contribution to the transfer of knowledge, ultimately increasing the entrepreneurial activity of students. Thus, training in this area is key to improving access for students to the labor market, increasing entrepreneurial intentions and promoting self-employment (Bae et al., 2014; Lanero et al., 2011; Sánchez, 2013). Furthermore, successful entrepreneurial education is considered key to dealing with the challenges seen in the world’s economies (Chiru et al., 2012). Therefore, the results are applicable to the development of both short- and long-term strategies, to promote entrepreneurial activity and develop increasingly solid and interconnected ecosystems, taking into account the role of the Entrepreneurial University as an engine of economic development, specifically facilitating the generation of entrepreneurial human capital (Audretsch, 2009).

In further research, it would be interesting to delve deeper into the process of competence acquisition, in order to determine the importance of the context in which competences develop, allowing differentiation between competences developed personally, academically, or professionally. Other variables could also be incorporated into the study, such as the entrepreneurial intention or the entrepreneurial activity, in order to identify the relationship between the development of competence, the entrepreneurial initiative, and real performance through business creation. Also important is the analysis of students’ characteristics in each of the groups. In this sense, future studies could integrate sociodemographic variables that provide information about the students that make up each of the profiles. This would provide more information to establish comparisons between groups, while at the same time enabling multivariate analyses focused on defining the explanatory variables that determine belonging to the profiles, such as gender, age, nationality, degree, educational level, or professional experience. Entrepreneurial education is also understood to be a key factor in this regard, therefore inclusion of this aspect in future studies would enable the analysis of differences in competence level between students who have received entrepreneurial training and those who have not. Comparisons between groups could help to determine the most important educational strategies and to provide information on the quality and utility of entrepreneurial training programs. Finally, further study on the characteristics, backgrounds, and process of competence acquisition of students belonging to the *top entrepreneurial profile* is important to improve our understanding of how competence develops for these more successful cases.

## Data Availability Statement

The raw data supporting the conclusions of this article will be made available by the authors, without undue reservation.

## Author Contributions

Both authors collaborated and contributed to the conception and design of the work, collected and analyzed the data, wrote the manuscript, performed the substantial contributions to revising the work critically, and contributed to the article and approved the submitted version.

## Conflict of Interest

The authors declare that the research was conducted in the absence of any commercial or financial relationships that could be construed as a potential conflict of interest.

## Annexes

**TABLE A1:** Classification of entrepreneurial competences and student assessment questionnaire (Morris et al., 2013).

Competence	Item code	Item description
Opportunity Recognition	p1a	I am an avid information seeker.
	p1b	I am always actively looking for new information.
	p1c	I often make novel connections and perceive new or emergent relationships between various pieces of information.
	p1d	I see links between seemingly unrelated pieces of information.
	p1e	I am good at “connecting dots.”
	p1f	I often see connections between previously unconnected domains of information.
Opportunity Assessment	p2a	I have a gut feeling for potential opportunities.
	p2b	I can distinguish between profitable opportunities and not so profitable opportunities.
	p2c	I have an extraordinary ability to smell profitable opportunities.
	p2d	I have a knack for telling high-value opportunities apart from low-value opportunities.
	p2e	When facing multiple opportunities, I am able to select the good ones.
Risk Management/Mitigation	p3a	My skills in recognizing and assessing risks are strong.
	p3b	There is not much the entrepreneur can do about risk.
	p3c	Risks cannot really be managed.
	p3d	I understand a lot about how to manage risks.
	p3e	Dealing with risk is a learned skill.
Conveying a compelling vision/seeing the future	p4a	I am always seeking new opportunities in my life.
	p4b	I believe in a bold and daring view of the future.
	p4c	I am able to paint an interesting picture of the future.
	p4d	The future is very hard to see or envision.
	p4e	I find it difficult to get others committed to my vision or dreams.
	p4f	I find that I am able to inspire others with my plans for the future.
Tenacity/Perseverance	p5a	New ideas and projects sometimes distract me from existing ones.
	p5b	My interests change from year to year.
	p5c	I have been obsessed with a certain idea or project for a short time but later lose interest.
	p5d	I have difficulty maintaining my focus on projects that take more than a few months to complete.
	p5e	I have achieved a goal that took years of work.
	p5f	I have overcome setbacks to conquer an important challenge.
	p5g	I finish whatever I begin.
	p5h	Setbacks don’t discourage me.
	p5i	I am a hard worker.
	p5j	I am diligent.
	p5k	I am a persistent person.
	p5l	I don’t let past failures hinder future performance.
	p5m	I don’t get easily frustrated when things don’t go my way.
	p5n	Nothing is more important than the achievement of my goals.
Creative Problem Solving/Imaginativeness	p6a	I demonstrate originality in my work.
	p6b	I am creative when asked to work with limited resources.
	p6c	I identify ways in which resources can be recombined to produce novel products.
	p6d	I find new uses for existing methods or equipment.
	p6e	I think outside of the box.
	p6f	I identify opportunities for new services/products.
	p6g	Freedom to be creative and original is extremely important to me.
Resource Leveraging/Bootstrapping	p7a	When I think about starting a venture, being able to access resources is far more important than actually owning and controlling those resources.
	p7b	It is important to me that the business owns all the necessary resources for its operations.
	p7c	The need for resources can be solved without any costs, for example by using resources that others control.
	p7d	Without sufficient savings or access to money, it is very hard to start a business.
	p7e	There is always a way to obtain a resource even if you cannot afford it.
	p7f	prefer to use well-planned and calculated market research tools when investigating the need and interest in my product/service.
	p7g	I prefer to use informal methods when investigating the need for or interest in my product/service (for example by asking people of my acquaintance, making my own observations etc.)
	p7h	When I am to realize a business opportunity I only invest as much as I can afford to lose.
	p7i	Mobilizing resources in unusual ways.
	p7j	Gaining leverage from limited resources
	p7k	Reducing your resource requirements (economize).
	p7l	Finding ways to actually create new resources, competences, technologies.
	p7m	Establishing strategic relationships based on reciprocity.
	p7n	Responding to challenges and tasks by redeploying resources in different ways.
	p7o	Using others people’s resources instead of your own.
Guerrilla actions	p8a	I am very comfortable thinking and acting in guerrilla ways.
	p8b	I could quickly identify three guerrilla ideas to help any start-up venture.
Value Creation with New Products, Services, Business Models	p9a	I am always asking questions.
	p9b	I am constantly asking questions to get to the root of the problem.
	p9c	Others sometimes get frustrated by the frequency of my questions.
	p9d	I often ask questions that challenge the status quo.
	p9e	I regularly ask questions that challenge others’ fundamental assumptions.
	p9f	I am constantly asking questions to understand why products and projects underperform.
	p9g	New business ideas often come to me when directly observing how people interact with products and services.
	p9h	I have a continuous flow of new business ideas that come through observing the world.
	p9i	I regularly observe customers’ use of products and services to get new ideas.
	p9j	By paying attention to everyday experiences, I often get new business ideas.
	p9k	I love to experiment to understand how things work and to create new ways of doing things.
	p9l	I frequently experiment to create new ways of doing things.
	p9m	I am adventurous, always looking for new experiences.
	p9n	I actively search for new ideas through experimenting.
	p9o	I have a history of taking things apart.
Ability to Maintain Focus yet Adapt	p10a	Once I have identified an approach for accomplishing a task, I find it very difficult to switch to a completely different approach.
	p10b	I find it easy to modify or change my ideas about how something should be done.
	p10c	Once I figure out something that works, I tend to resist changes to that particular approach.
	p10d	I tend to look for the right answer, rather than realize there might be multiple ways to get to an end result.
	p10e	It is easy for me to modify my approach to a task if the situation calls for it.
	p10f	When I feel that my approach to a given task is not working, I find it quite easy to change to another approach.
Resilience	p11a	I actively look for ways to replace the losses I encounter in life.
	p11b	I look for creative ways to alter difficult situations.
	p11c	I believe that I can grow in positive ways by dealing with difficult situations.
	p11d	Regardless of what happens to me, I believe I can control my reaction to it.
	p11e	I only set goals which I know I can reach without the help of others.
	p11f	When I need help, I don’t hesitate to ask a friend to help.
	p11g	I hesitate to ask others to help me.
	p11h	My friends and family frequently don’t live up to my expectations of how they should act.
	p11i	I really resent anyone telling me what to do.
Self-Efficacy	p12a	Entrepreneurs are not really able to create and shape their own markets.
	p12b	As regards competing in the marketplace, the entrepreneur is the victim of forces he/she cannot control.
	p12c	There is little point in engaging in detailed analyses and planning, because events will occur that I cannot control.
	p12d	I can shape whatever environment in which I find myself operating.
Networking/Social Skills	p13a	Given professional contacts a phone call to keep in touch.
	p13b	Sent thank you notes or gifts to others who have helped you professionally in your work, school or career.
	p13c	Asked a business professional unrelated to you to serve as a reference
	p13d	Sent e-mails, cards or other communications to keep in touch with professional contacts.
	p13e	Gone to lunch with persons who can help you professionally.
	p13f	Participated in social gatherings with people that you work with in a non-campus job.
	p13g	Attended social functions for purposes of building professional relationships.
	p13h	Gone to lunch with a boss or supervisor.
	p13i	Attended meetings of professional-related organizations.
	p13j	Attended professional seminars or workshops.
	p13k	Attended meetings of civic and social groups, clubs and so forth.
	p13l	Given professional seminars, workshops or public speech.
	p13m	Attended conferences or trade shows.
	p13n	Participated in church work projects.
	p13o	Participated in church social functions.
	p13p	Participated in community projects.
	p13q	Served on a community board, committee or task force.

*All items measured with a five-point Likert scale. Items p7i-p70 (Not at all comfort–Very comfort). Items p13a-p13q (Not at all–Quite frequently). Other items (strongly disagree-strongly agree).*

**TABLE A2:** Factor loadings (varimax rotation); *n* = 1081; n° of factors: 12.

Variable	Factor 1	Factor 2	Factor 3	Factor 4	Factor 5	Factor 6	Factor 7	Factor 8	Factor 9	Factor 10	Factor 11	Factor 12
p1a									0.4011			
p1b									0.5216			
p1c									0.5918			
p1d									0.5609			
p1e		0.3372							0.5209			
p1f		0.3095				0.3875			0.3399			
p2a						0.6160						
p2b					0.3663	0.5852						
p2c						0.7088						
p2d						0.6150						
p2e						0.4769						
p3a					0.5579							
p3b					0.3001							
p3c					0.3073							
p3d					0.3224							
p4a						0.3344						
p4b						0.3136						
p5a										0.5831		
p5b										0.6561		
p5c										0.6811		
p5f								0.4942				
p5g								0.4723				
p5h								0.6515				
p5i								0.6199				
p5j								0.5984				
p6a		0.6969										
p6b		0.6983										
p6c		0.6572										
p6d		0.6237										
p6e		0.6554										
p6f		0.5533										
p7i							0.5239					
p7j							0.5062					
p7k							0.5147					
p7l							0.6490					
p7m							0.5476					
p7n					0.3585		0.3410					
p8a		0.3510										
p8b				0.4925								
p9a				0.7024								
p9b				0.6339								
p9c				0.7268								
p9d				0.6928								
p9e				0.5965								
p9f				0.3684								
p9g		0.3942	0.5311									
p9h		0.3356	0.6053									
p9i		0.3734	0.5792									
p9j		0.3055	0.5888									
p9k			0.6314									
p9l			0.5859									
p9m			0.6490									
p9n			0.3878									
p10e												0.4592
p10f												0.5193
p11a												0.5258
p11b												0.3931
p11c												0.4627
p12b					0.5619							
p12d	0.4115				0.3397							
p13a	0.5811											
p13b	0.4592											
p13c	0.5886											
p13d	0.6904											
p13e	0.6520											
p13f	0.6713											
p13g	0.6457											
p13h	0.6284											
p13i	0.5933											
p13j	0.5045											
p13k	0.4509											
p13l	0.4524											
p13m											0.4874	
p13n											0.7455	
p13o											0.6108	
p13p											0.4031	
p13q											0.3002	

*Blanks represent factor loading <0.3.*
